# Naringin Attenuates Steatosis, Oxidative Stress, Inflammation, and Fibrosis in MASH: Network Pharmacology and In Vivo Study

**DOI:** 10.3390/biom16050651

**Published:** 2026-04-27

**Authors:** Ji-Han Kim, Seung-Hoon Yoo, Yeon-Joo Yoo, Byung-Cheol Lee

**Affiliations:** Department of Clinical Korean Medicine, Graduate School, Kyung Hee University, 26 Kyungheedae-ro, Dongdaemun-gu, Seoul 02447, Republic of Korea; ilpc21@khu.ac.kr (J.-H.K.); ysh9306@khu.ac.kr (S.-H.Y.); alice98113@khu.ac.kr (Y.-J.Y.)

**Keywords:** metabolic dysfunction–associated steatohepatitis, naringin, network pharmacology, hepatic steatosis, oxidative stress, inflammation, fibrosis

## Abstract

Metabolic dysfunction-associated steatohepatitis (MASH) is a progressive stage of metabolic dysfunction-associated steatotic liver disease characterized by lipid dysregulation, oxidative stress, inflammation, and fibrosis. Because these processes occur simultaneously, compounds targeting multiple pathways may offer therapeutic benefit. Naringin, a citrus-derived flavonoid, has reported antioxidant and anti-inflammatory properties, but its integrated effects in MASH remain unclear. In this study, the effects of naringin were evaluated using combined in silico analysis and in vivo experiments. Network pharmacology and molecular docking predicted targets related to lipid metabolism, oxidative stress, inflammation, and fibrosis, which were validated in a methionine- and choline-deficient diet-induced mouse model. Naringin reduced hepatic lipid accumulation and improved serum AST and ALT levels. It modulated oxidative stress-related genes, attenuated inflammatory responses, and reduced fibrogenic markers. Naringin also decreased Ly6Chigh inflammatory monocytes and Kupffer cell activation, and reduced hypothalamic microglial activation. These findings suggest that naringin exerts multi-target effects across hepatic, systemic, and central pathways, supporting its potential as a therapeutic candidate for MASH.

## 1. Introduction

Metabolic dysfunction-associated steatohepatitis (MASH), the progressive inflammatory form of metabolic dysfunction-associated steatotic liver disease (MASLD), has emerged as a major cause of chronic liver disease worldwide and is increasingly recognized as a growing indication for liver transplantation [1]. MASLD, previously referred to as nonalcoholic fatty liver disease (NAFLD), encompasses a spectrum of hepatic disorders associated with metabolic dysfunction. MASH is characterized by hepatic steatosis, hepatocellular injury, inflammation, and fibrosis, and can progress to cirrhosis and hepatocellular carcinoma, thereby contributing substantially to global morbidity and mortality [2,3,4].

The pathogenesis of MASH is driven by a complex interplay among lipid dysregulation, oxidative stress, inflammatory signaling, and activation of hepatic stellate cells, which collectively promote progressive liver fibrosis [5]. These processes are further influenced by both metabolic and genetic determinants, highlighting the multifactorial nature of disease development and progression [6]. Consequently, therapeutic strategies capable of targeting interconnected metabolic and inflammatory pathways are required to effectively prevent hepatic injury and fibrogenesis.

Despite extensive investigation into pharmacological interventions, no therapy has yet demonstrated sufficient efficacy and safety to establish a definitive standard treatment for MASLD/MASH [7,8]. Given the integrated metabolic and immune disturbances underlying disease progression, single-target approaches may be insufficient to halt hepatic injury and fibrosis. Accordingly, multitarget strategies that simultaneously modulate lipid metabolism, inflammatory signaling, and fibrotic activation have gained increasing attention [9]. In this context, natural products and plant-derived compounds represent promising therapeutic candidates due to their pleiotropic biological activities and relatively favorable safety profiles [10,11].

Beyond its hepatic manifestations, increasing evidence suggests that steatotic liver disease is closely linked to systemic immune activation and neuroinflammatory responses within the central nervous system [2]. Liver-derived inflammatory mediators and circulating immune cells can influence blood–brain barrier integrity and activate hypothalamic microglia, thereby affecting appetite regulation, energy homeostasis, and insulin sensitivity [12,13,14]. This emerging concept of the liver–systemic–brain axis highlights the importance of integrated therapeutic strategies capable of simultaneously modulating hepatic injury, peripheral immune dysregulation, and central neuroinflammation [15].

Citrus flavonoids have attracted considerable attention as potential therapeutic candidates for metabolic liver diseases due to their antioxidant, anti-inflammatory, antifibrotic, and lipid-modulating properties [16]. Naringin, a major flavanone glycoside abundant in citrus fruits, particularly Aurantii Fructus Immaturus, is metabolized in vivo to its aglycone form naringenin, which exhibits higher bioavailability and biological activity [17]. Previous studies have reported that naringin and naringenin exert hepatoprotective effects in experimental models by regulating lipid metabolism, β-oxidation, oxidative stress responses, inflammatory signaling pathways, and fibrogenesis [18]. These effects are closely associated with the regulation of metabolic inflammation and hepatic lipid metabolism, processes that play central roles in the pathogenesis of MASLD/MASH [17,19,20]. However, the integrated multi-target mechanisms of naringin in the context of MASH, particularly its potential effects on systemic immune regulation and neuroinflammatory alterations, remain incompletely understood.

The methionine- and choline-deficient (MCD) diet model is widely used to induce hepatic steatosis, oxidative stress, inflammation, and fibrosis through impaired very-low-density lipoprotein (VLDL) synthesis and enhanced oxidative injury [19,21]. Although the MCD model does not fully reproduce the metabolic phenotype associated with obesity-driven MASLD, it reliably recapitulates the inflammatory and fibrotic features of MASH, making it suitable for evaluating anti-inflammatory and antifibrotic therapeutic interventions [19].

In the present study, we integrated network pharmacology, molecular docking, and an MCD diet-induced mouse model to investigate the multitarget mechanisms of naringin in MASH. The hypothalamus is a key regulatory center for metabolic homeostasis, and increasing evidence suggests that hypothalamic neuroinflammation contributes to the development of metabolic disorders [22]. Based on this rationale, we examined the regulatory effects of naringin on hepatic lipid accumulation, oxidative stress, inflammatory and fibrotic pathways, peripheral immune cell profiles, and hypothalamic microglial activation. By analyzing these interconnected processes, we aimed to determine whether naringin exerts coordinated regulatory effects across hepatic, systemic immune, and hypothalamic pathways and to evaluate its potential as a multitarget modulator of these interconnected systems in MASH.

## 2. Materials and Methods

### 2.1. In Silico Analysis

Naringin, a major bioactive compound of *Aurantii Fructus Immaturus* (Zhi Shi), was selected as the candidate molecule for analysis. Pharmacokinetic properties including oral bioavailability (OB), Caco-2 permeability, and drug-likeness (DL) were obtained from the Traditional Chinese Medicine Systems Pharmacology (TCMSP) database.

Although naringin did not fully meet the conventional ADME screening criteria (OB > 30%, DL > 0.18, Caco-2 > −0.4), it was included because it is present in high amounts in Zhi Shi and is metabolized in vivo into the active aglycone naringenin, which shows higher bioavailability and pharmacological activity [23].

Potential molecular targets of naringin were predicted using SwissTargetPrediction (version 2019) based on the SMILES structure obtained from PubChem. Genes related to metabolic dysfunction-associated steatohepatitis (MASH) were collected from the GeneCards database using the keyword “metabolic dysfunction–associated steatohepatitis”. Overlapping targets were identified using Venny 2.1.

Protein–protein interaction (PPI) networks were constructed using the STRING database and visualized using Cytoscape software (version 3.10.4). Functional enrichment analysis, including Gene Ontology (GO) analysis for biological process (BP), cellular component (CC), and molecular function (MF), and Kyoto Encyclopedia of Genes and Genomes (KEGG) pathway analysis, was performed using the DAVID Bioinformatics Resources (version 6.8). 

Genes related to lipid metabolism, oxidative stress, inflammation, and fibrosis were selected for molecular docking analysis. Docking simulations were performed using the PyRx virtual screening tool. The three-dimensional structure of naringin was obtained from PubChem, and crystal structures of target proteins were downloaded from the RCSB Protein Data Bank. Protein structures were preprocessed using BIOVIA Discovery Studio Visualizer (version 24.1.0.23298). Binding affinity was calculated, and docking poses were visualized using PyMOL (version 2.5.8).

### 2.2. Animals and Experimental Design

Naringin (Tokyo Chemical Industry, Tokyo, Japan) was dissolved in normal saline immediately before administration. Twenty-five male C57BL/6 mice (6 weeks old) were obtained from Central Lab Animal, Inc., Seoul, Republic of Korea and maintained under controlled conditions (40–70% humidity, 12-h light/dark cycle) with free access to food and water. All procedures were approved by the Institutional Animal Care and Use Committee of Kyung Hee Medical Center (KHMC-IACUC 2024-030).

After one week of acclimatization on a normal control diet, baseline body weight was recorded, and mice were randomly divided into five groups (*n* = 5 per group): NC (normal diet), MCD (methionine–choline-deficient diet), NAR 100 (MCD + naringin 100 mg/kg/day), NAR 200 (MCD + naringin 200 mg/kg/day), and PIO (MCD + pioglitazone 30 mg/kg/day).

Pioglitazone, a PPARγ agonist known to ameliorate steatohepatitis in experimental models, was used as a positive control [24].

During the subsequent 4-week experimental period, mice in the MCD, NAR 100, NAR 200, and PIO groups were fed the MCD diet, while the NC group received a normal control diet. Drug administration began at the start of the MCD feeding period and continued once daily by oral gavage throughout the 4-week experiment. NC and MCD groups received the same volume of saline.

Body weight was measured at baseline and weekly during the 4-week feeding period. Food intake was recorded daily and converted to caloric intake per mouse (kcal/day). At the end of the 4-week feeding period, mice were anesthetized, and blood and tissues were collected. Liver and epididymal adipose tissues were excised and weighed.

### 2.3. Biochemical and Histological Analysis

Blood samples were collected by cardiac puncture, centrifuged, and serum was stored at −40 °C until analysis. Serum AST, ALT, total cholesterol (TC), HDL, LDL, triglyceride (TG), non-esterified fatty acid (NEFA), and phospholipid levels were measured using standard enzymatic methods.

Liver tissues were fixed in 10% neutral-buffered formalin, embedded in paraffin, and sectioned at 4 μm thickness. Sections were stained with hematoxylin and eosin (H&E) to evaluate steatosis and inflammation, and Sirius Red staining was performed to assess collagen deposition.

Images were obtained under identical conditions, and quantitative analysis of stained areas was performed using ImageJ software (version 1.53e) 

### 2.4. RNA Extraction and Quantitative Real-Time PCR

Liver tissues were rapidly frozen in liquid nitrogen and stored at −70 °C. Total RNA was extracted using the Mini RNA Isolation II™ kit (Zymo Research, Irvine, CA, USA), and cDNA was synthesized using the Advantage RT for PCR kit (Clontech, Mountain View, CA, USA).

Quantitative real-time PCR was performed using SYBR Green on a 7900HT Fast Real-Time PCR System (Applied Biosystems, Carlsbad, CA, USA). Relative gene expression was calculated using the 2−ΔΔCt method with GAPDH as the internal control.

Genes for in vivo analysis were selected based on both predicted targets identified through network pharmacology and their relevance to key pathological processes in the MCD model, rather than a direct one-to-one mapping from in silico predictions. Specifically, representative genes associated with major pathological features of steatohepatitis were included, encompassing lipogenesis (*Srebf1, Fasn*, *Ppara*, *Cpt1a*), oxidative stress (*Sod2*, *Cat*, *Cyp2e1*), inflammation (*Adgre1*, *Ccl2*, *Tnf*, *Il6*, *Il10*), and fibrosis (*Col3a1*, *Acta2*, *Tgfb1*, *Timp1*).

### 2.5. Flow Cytometry

Peripheral blood was collected into EDTA-treated tubes. After Fc receptor blocking, cells were stained with fluorochrome-conjugated monoclonal antibodies against CD45, CD11b, and Ly6C. Peripheral monocyte subsets were analyzed by flow cytometry, and Ly6Chigh and Ly6Clow monocytes were gated within the CD45^+^CD11b^+^ population using a FACSCalibur flow cytometer (BD Biosciences, San Jose, CA, USA). Data were analyzed with FlowJo software (Ashland, OR, USA).

For hepatic immune cell analysis, liver tissues were digested with collagenase and DNase I to obtain single-cell suspensions. Cells were stained with antibodies against CD45, F4/80, and CD11b. Kupffer cells were defined as CD45^+^F4/80^+^ and CD45^+^F4/80^+^CD11b^+^ populations and quantified by flow cytometry.

### 2.6. Brain Immunohistochemistry

Brain tissues were fixed in 10% neutral-buffered formalin, embedded in paraffin, and sectioned at 3–4 μm thickness. After deparaffinization and antigen retrieval using citrate buffer, sections were incubated with primary antibodies against Iba-1 and GFAP, followed by appropriate secondary antibodies. Immunoreactivity was visualized using DAB chromogen, and sections were counterstained with hematoxylin.

Images were obtained under identical exposure conditions. Immunoreactivity of Iba-1-positive microglia and GFAP-positive astrocytes was quantified using ImageJ software. Negative controls were prepared without primary antibody.

### 2.7. Statistical Analysis

Statistical analyses were performed using GraphPad Prism 8.0 (GraphPad Software, USA). Results are expressed as mean ± standard error of the mean (SEM). Differences among groups were analyzed using one-way analysis of variance (ANOVA) followed by Tukey’s post hoc test. A *p* value < 0.05 was considered statistically significant.

Predefined comparisons were performed between NC and MCD groups, and treatment groups (NAR 100, NAR 200, and PIO) were compared with the MCD group.

## 3. Results

### 3.1. In Silico Pharmacokinetics, Target Identification, and Network-Based Mechanistic Prediction

**Figure 1:**
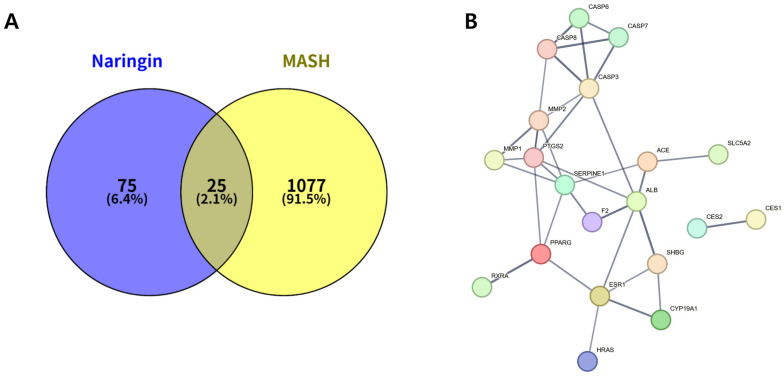
Target overlap and interaction network between naringin (NAR) and metabolic dysfunction–associated steatohepatitis (MASH). (**A**) Venn diagram showing the overlapping genes between naringin-related targets and MASH-associated genes. (**B**) STRING-based protein–protein interaction (PPI) network of the overlapping targets.

In silico ADME analysis indicated low oral bioavailability (6.92%) and low Caco-2 permeability (−1.99) for naringin, while drug-likeness met the commonly used threshold (DL = 0.78). To explore potential mechanistic relevance in MASH, SwissTargetPrediction and GeneCards analyses were performed, and 25 overlapping genes between naringin-related targets (*n* = 100) and MASH-associated genes (*n* = 1102) were identified (Figure 1A).

Among these, 20 genes were represented in the STRING-based PPI network. The PPI network constructed in STRING consisted of 20 nodes and 32 edges (Figure 1B), and hub analysis highlighted PPARG, PTGS2, SERPINE1, CASP3, MMP2, ESR1, CASP8, SHBG, and ALB as highly connected nodes.

**Figure 2:**
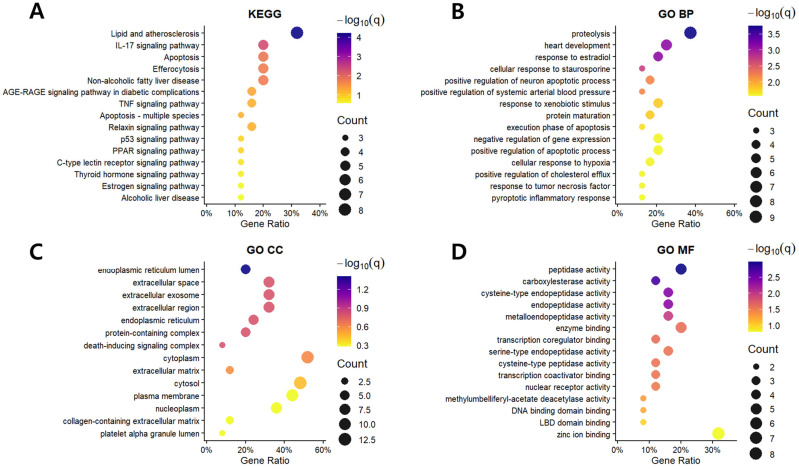
KEGG and Gene Ontology (GO) enrichment analysis of the overlapping target genes between naringin and MASH. (**A**) KEGG pathways; (**B**) GO biological process (BP); (**C**) GO cellular component (CC); (**D**) GO molecular function (MF). Dot size corresponds to the number of enriched genes, and dot color represents the −log10 (q-value).

KEGG enrichment analysis demonstrated significant enrichment of pathways related to lipid metabolism, inflammation, apoptosis, and fibrotic remodeling (Figure 2A). GO enrichment analysis further categorized the targets into biological process (BP), cellular component (CC), and molecular function (MF) domains (Figure 2B–D). Based on hubness and pathway relevance, docking analysis was conducted for hub genes and additional biologically relevant targets, reflecting both network centrality and their involvement in key pathological processes of MASH. Thus, the target selection for docking was not limited to a direct subset of the PPI network but was refined based on biological relevance and functional representation.

**Figure 3:**
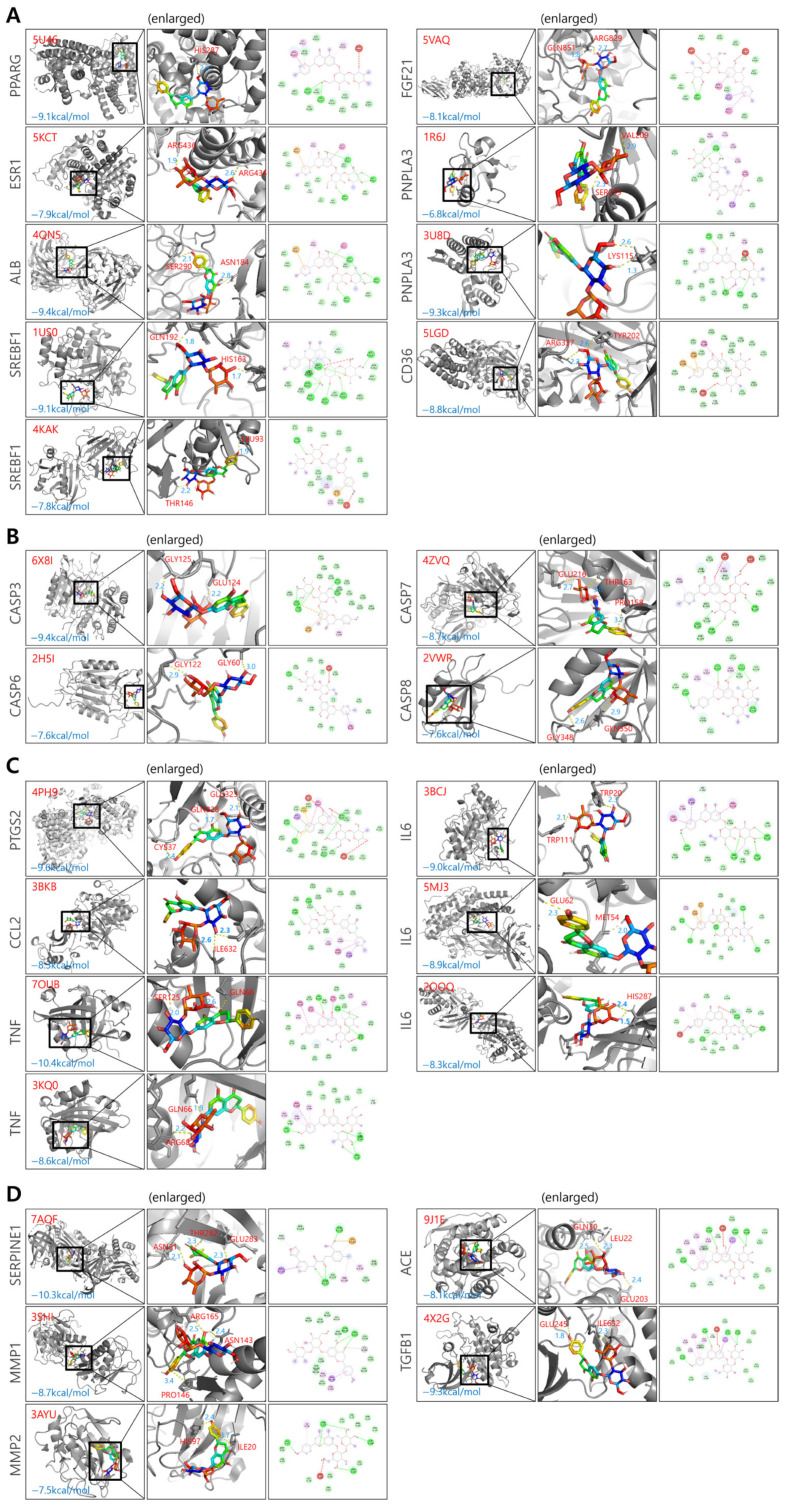
Molecular docking analysis of naringin with representative MASH-related target proteins. (**A**) Lipid metabolism-related targets, including PPARG, ESR1, ALB, SREBF1, FGF21, PNPLA3, and CD36. (**B**) Cellular stress-related targets, including CASP3, CASP6, CASP7 and CASP8. (**C**) Inflammation-related targets, including PTGS2, CCL2, IL6, and TNF. (**D**) Fibrosis-related targets, including SERPINE1, MMP1, MMP2, ACE and TGFB1. For each target, the left panel shows the overall three-dimensional (3D) binding conformation, the middle panel presents an enlarged 3D view of the binding interactions, and the right panel illustrates the two-dimensional (2D) interaction map. Binding energies (kcal/mol) are indicated for each complex. Colors indicate different types of molecular interactions: green circles represent van der Waals interactions; bright green dashed lines indicate conventional hydrogen bonds; red dashed lines indicate unfavorable acceptor–acceptor interactions; pink dashed lines indicate π–π stacking interactions; light pink dashed lines indicate π–alkyl interactions.

Naringin showed broadly favorable docking scores, with representative binding conformations illustrated in Figure 3. A summary of the docking results, including binding affinities for each target, is provided in Table 1. Multiple PDB structures were used for each target to account for structural variability and improve docking reliability. These structures may differ in conformational states, resolution, co-crystallized ligands, and binding site accessibility. As a result, even for the same protein target, the binding affinity (docking energy) of naringin may vary depending on the specific protein structure used.

### 3.2. Effects of Naringin on Body Weight, Energy Intake, and Organ Weights

**Figure 4:**
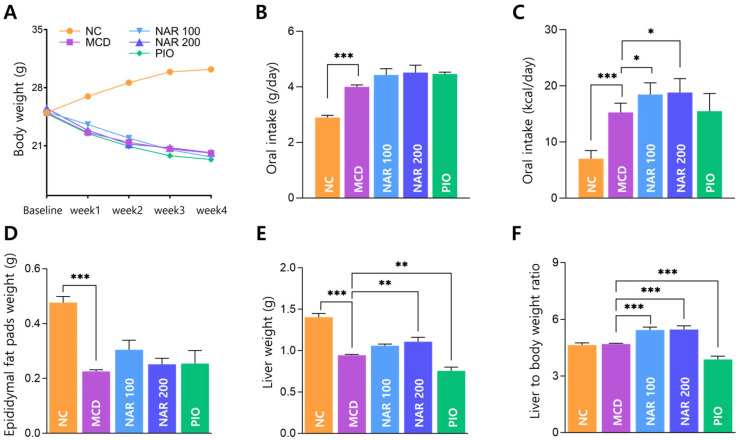
Effects of naringin on body weight, energy intake, and organ weights in MCD-fed mice. (**A**) Body weight changes from baseline (after 1 week of acclimation) to Week 4 of MCD feeding with treatment. (**B**) Daily food intake (g/mouse/day). (**C**) Daily caloric intake (kcal/mouse/day). (**D**) Epididymal fat pad weight at sacrifice. (**E**) Liver weight at sacrifice. (**F**) Liver-to-body weight ratio. Results are expressed as mean ± SEM. * *p* < 0.05, ** *p* < 0.01, *** *p* < 0.001. MCD was compared with NC, and treatment groups (NAR 100, NAR 200, and PIO) were compared with MCD.

After one week of acclimation, baseline body weights were comparable among groups. During the subsequent 4-week experimental period, MCD-fed mice exhibited progressive weight loss compared with the NC group, and neither naringin nor pioglitazone significantly altered body weight relative to the MCD group (Figure 4A).

MCD feeding increased daily food intake and caloric intake compared with NC, and caloric intake was higher in naringin-treated groups relative to MCD (Figure 4B,C).

At sacrifice, epididymal fat pad and liver weights were significantly reduced in the MCD group compared with NC (Figure 4D,E). Naringin attenuated the reduction in liver weight, with a significant increase observed in the NAR 200 group versus MCD, whereas pioglitazone further decreased liver weight (Figure 4E). The liver-to-body weight ratio was increased in both naringin-treated groups and decreased in the pioglitazone group (Figure 4F). The lack of a marked change in the MCD group may reflect concurrent reductions in both body weight and liver weight. Although absolute liver weight was reduced in the MCD model, the relatively higher ratio observed in the naringin-treated groups may reflect a relative preservation of liver mass compared to body weight, rather than aggravated hepatic injury.

### 3.3. Naringin Attenuates Hepatic Steatosis and Fibrosis in MCD-Induced MASH

**Figure 5:**
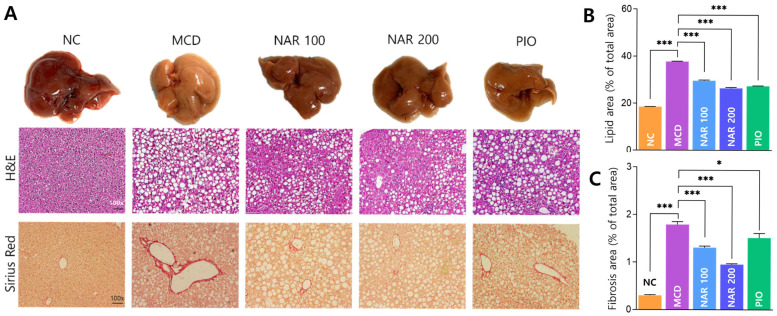
Naringin attenuates hepatic steatosis and fibrosis in MCD-induced MASH. (**A**) Representative gross liver images and histological staining (H&E and Sirius Red). (**B**) Quantification of lipid droplet area. (**C**) Quantification of fibrotic area. Results are expressed as mean ± SEM. * *p* < 0.05, *** *p* < 0.001. MCD was compared with NC, and treatment groups (NAR 100, NAR 200, and PIO) were compared with MCD. Scale bar = 100 μm.

Gross inspection revealed marked liver shrinkage in MCD-fed mice, which was partially restored by naringin treatment (Figure 5A). H&E staining showed extensive vacuolation and lipid accumulation in the MCD group, with a significant increase in lipid area compared with NC. Both naringin doses significantly reduced lipid accumulation, with greater improvement at 200 mg/kg; pioglitazone similarly reduced lipid area (Figure 5B). Sirius Red staining demonstrated substantial collagen deposition in the MCD group. Naringin significantly suppressed fibrosis, and the high-dose group showed the greatest reduction, whereas pioglitazone produced a smaller improvement (Figure 5C).

### 3.4. Effects of Naringin on Serum Biochemical Markers and Lipid Profiles

**Figure 6:**
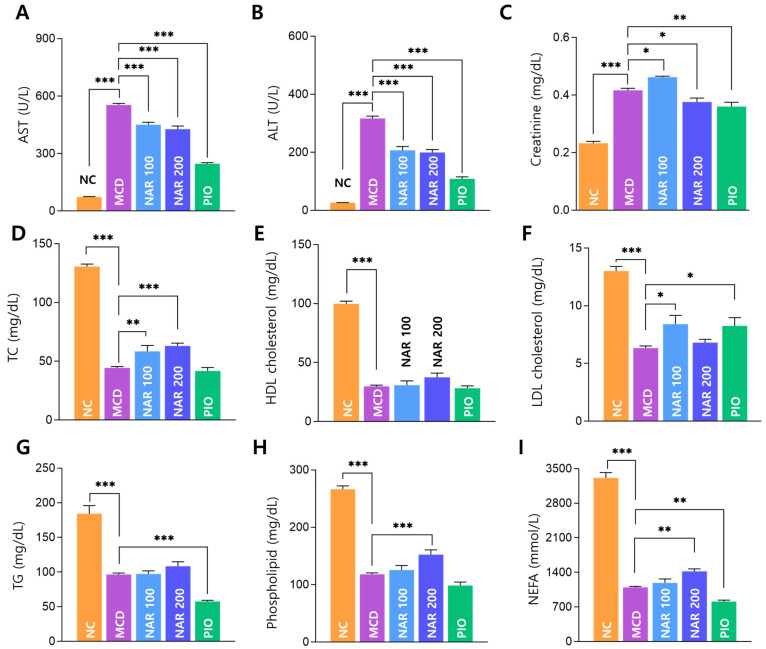
Effects of naringin on serum biochemical markers and lipid profiles in MCD-induced MASH. (**A**–**C**) Serum AST, ALT, and creatinine. (**D**–**I**) Serum lipid parameters (TC, HDL-C, LDL-C, TG, phospholipids, and NEFA). Results are expressed as mean ± SEM. * *p* < 0.05, ** *p* < 0.01, *** *p* < 0.001. MCD was compared with NC, and treatment groups (NAR 100, NAR 200, and PIO) were compared with MCD.

MCD feeding markedly elevated serum AST and ALT levels compared with NC, and both naringin doses significantly reduced AST and ALT relative to MCD (Figure 6A,B). Creatinine levels were increased in MCD-fed mice, and a significant reduction was observed in the naringin and pioglitazone groups (Figure 6C).

Circulating lipid parameters (TC, HDL-C, LDL-C, TG, phospholipids, and NEFA) were significantly decreased by MCD feeding compared with NC. Naringin showed partial changes in selected lipid parameters, but the effects were not consistently dose-dependent, whereas pioglitazone exhibited a different metabolic profile compared with the MCD group (Figure 6D–I).

### 3.5. Naringin Modulates Peripheral and Hepatic Immune Cell Profiles

**Figure 7:**
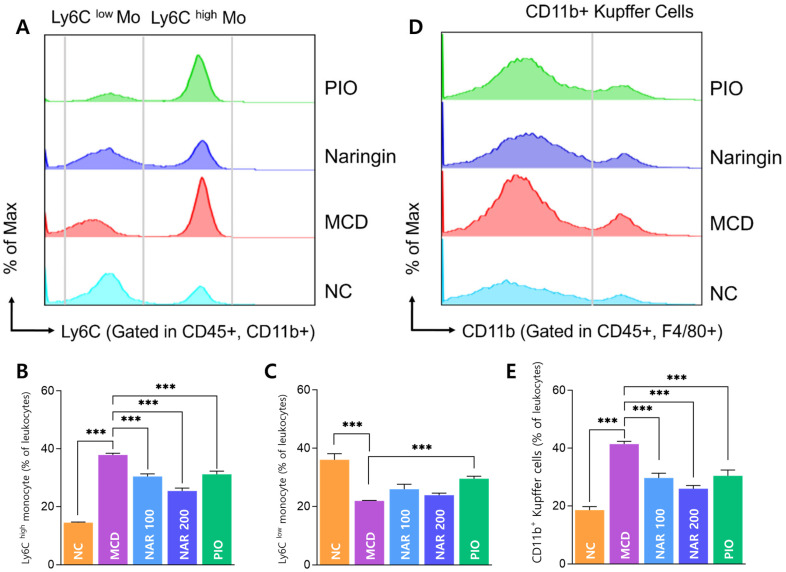
Effects of naringin on circulating Ly6C monocyte subsets and hepatic CD11b^+^ Kupffer cells. (**A**) Representative flow cytometry analysis of Ly6C^high^ and Ly6C^low^ monocyte subsets gated in CD45^+^CD11b^+^ cells (NAR 200 group). (**B**,**C**) Quantification of Ly6C^high^ and Ly6C^low^ monocyte populations. (**D**) Representative flow cytometry analysis of hepatic CD11b^+^ Kupffer cells gated in CD45^+^F4/80^+^ cells (NAR 200 group). (**E**) Quantification of CD11b^+^ Kupffer cells. Results are expressed as mean ± SEM. *** *p* < 0.001. MCD was compared with NC, and treatment groups (NAR 100, NAR 200, and PIO) were compared with MCD.

Flow cytometry revealed that MCD feeding significantly increased circulating Ly6C^high^ monocytes and decreased Ly6C^low^ monocytes compared with NC. Naringin significantly reduced Ly6C^high^ monocyte expansion in a dose-dependent manner (Figure 7A–C). Representative flow cytometry plots in Figure 7A correspond to the NAR 200 mg/kg group. Hepatic CD11b^+^ Kupffer cells were also elevated in the MCD group and were significantly reduced by both naringin doses (Figure 7D,E), and the representative plots in Figure 7D correspond to the NAR 200 mg/kg group.

### 3.6. Effects of Naringin on Hepatic Gene Expression Programs

**Figure 8:**
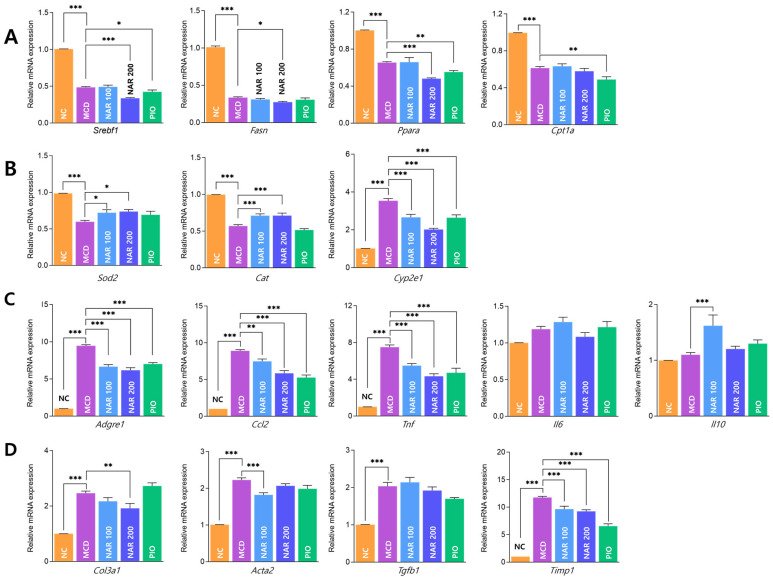
Effects of naringin on hepatic gene expression profiles in MCD-induced MASH mice. (**A**–**D**) Hepatic mRNA expression levels of genes involved in lipogenesis (*Srebf1*, *Fasn*, *Ppara*, *Cpt1a*) (**A**), oxidative stress (*Sod2*, *Cat*, *Cyp2e1*) (**B**), inflammation (*Adgre1*, *Ccl2*, *Tnf*, *Il6*, *Il10*) (**C**), and fibrosis (*Col3a1*, *Acta2*, *Tgfb1*, *Timp1*) (**D**). Results are expressed as mean ± SEM. * *p* < 0.05, ** *p* < 0.01, *** *p* < 0.001. MCD was compared with NC, and treatment groups (NAR 100, NAR 200, and PIO) were compared with MCD.

MCD feeding significantly altered hepatic transcriptional programs related to lipid metabolism, oxidative stress, inflammation, and fibrosis (Figure 8A–D). Naringin showed limited effects on lipid metabolism—related genes, with no consistent restoration of *Srebf1*, *Fasn*, *Ppara*, or *Cpt1a* expression (Figure 8A). Antioxidant-related genes (*Sod2* and *Cat*) were increased, while *Cyp2e1* was reduced in a dose-dependent manner (Figure 8B). Naringin significantly downregulated inflammatory mediators (*Adgre1*, *Ccl2*, and *Tnf*) (Figure 8C). Fibrosis-related genes were also reduced, with *Col3a1* decreased at 200 mg/kg, *Acta2* decreased at 100 mg/kg, and *Timp1* reduced at both doses (Figure 8D).

### 3.7. Naringin Reduces Hypothalamic Microglial Activation

**Figure 9:**
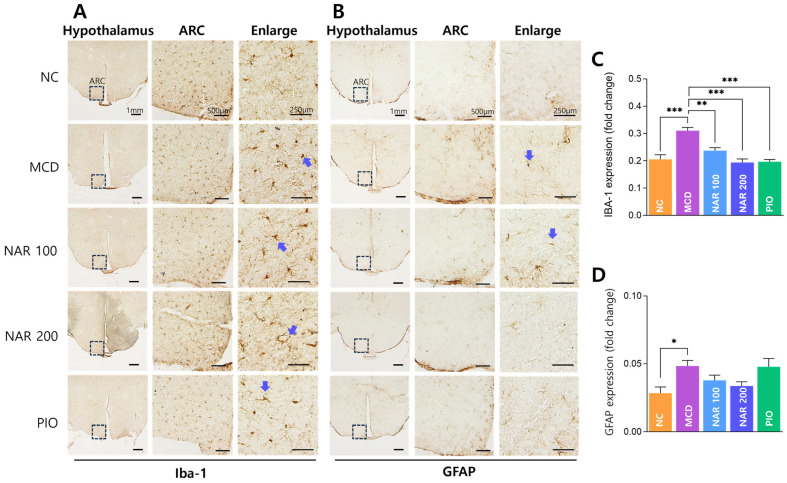
Effects of naringin on hypothalamic microglial and astrocyte activation in the arcuate nucleus (ARC). (**A**) Representative immunohistochemical staining of Iba-1 in the hypothalamic ARC. (**B**) Representative immunohistochemical staining of GFAP in the hypothalamic ARC. Low-magnification images (1 mm) indicate the anatomical location of the ARC, while higher-magnification images (500 μm and 250 μm) show the distribution of immunopositive cells. Arrows indicate representative immunopositive cells (Iba-1–positive microglia and GFAP-positive astrocytes). (**C**) Quantification of Iba-1 immunoreactivity. (**D**) Quantification of GFAP immunoreactivity. Results are expressed as mean ± SEM. * *p* < 0.05, ** *p* < 0.01, *** *p* < 0.001. MCD was compared with NC, and treatment groups (NAR 100, NAR 200, and PIO) were compared with MCD. Scale bars: 1 mm, 500 μm, and 250 μm.

In the hypothalamic arcuate nucleus (ARC), immunohistochemical observation showed increased microglial activation in the MCD group, which was attenuated after naringin administration (Figure 9A). Although morphological changes were not prominent, quantitative analysis confirmed that hypothalamic Iba-1 immunoreactivity was significantly increased in the MCD group compared with the NC group and was significantly reduced in both naringin-treated groups (Figure 9C). GFAP immunoreactivity was also increased in the MCD group and tended to decrease after naringin treatment, although the change did not reach statistical significance (Figure 9D). Representative GFAP staining images are shown in Figure 9B.

## 4. Discussion

In the present study, an integrated approach combining network pharmacology, molecular docking, and experimental validation was used to evaluate the multi-target regulatory potential of naringin in metabolic dysfunction-associated steatohepatitis. The results indicate that naringin exerts protective effects not through a single molecular mechanism but by simultaneously modulating lipid metabolism, oxidative stress, inflammatory signaling, immune cell activation, and fibrotic remodeling.

Network pharmacology analysis identified hub targets including ESR1, PTGS2, MMP2, and CASP3, which are known to be involved in inflammatory signaling, apoptosis, extracellular matrix regulation, and systemic metabolic control [25,26,27]. Several of these targets are closely associated with lipid metabolism, inflammatory pathways, and cell death signaling, suggesting that naringin may act at multiple stages of disease progression rather than through a single pathway.

Abnormal lipid metabolism is considered a primary initiating factor in the development of steatohepatitis [28]. Excessive hepatic lipid accumulation induces lipotoxicity and mitochondrial dysfunction, which subsequently promote oxidative stress, immune activation, and fibrotic responses during disease progression [29,30]. In the present study, network pharmacology analysis revealed significant enrichment of lipid metabolism-related pathways, including fatty acid metabolism and PPAR signaling. Molecular docking further suggested that naringin may interact with key lipid-regulating targets such as PPARG, SREBF1, CD36, FGF21, and PNPLA3. In vivo, changes in the expression of *Srebf1*, *Fasn*, and *Ppara* were observed; however, these changes did not represent a consistent restoration toward normal levels. This inconsistency may reflect the limitations of the MCD model, which does not fully recapitulate key metabolic features of human MASLD/MASH, such as obesity and insulin resistance [31]. Accordingly, the relatively modest or inconsistent effects observed in lipid metabolism-related genes (*Srebf1*, *Fasn*, *Ppara*, and *Cpt1a*) should be interpreted with caution.

Oxidative stress is a central pathogenic factor in the progression of steatohepatitis and is closely associated with excessive lipid accumulation and impaired lipid handling in hepatocytes. Increased lipid burden promotes the generation of reactive oxygen species, leading to cellular damage and subsequent activation of inflammatory signaling pathways. This oxidative stress enhances the expression of inflammatory cytokines and chemokines and promotes the activation of inflammatory responses. Network pharmacology analysis identified targets related to apoptosis and oxidative injury, including CASP3, CASP6, CASP7, and CASP8, suggesting that oxidative stress-related cell damage may play an important role in disease progression. In addition, in vivo experiments showed altered expression of oxidative stress-related genes such as *Sod2*, *Cat*, and *Cyp2e1*, supporting the possibility that naringin modulates the balance between oxidative injury and antioxidant defense mechanisms in the liver. These findings suggest that regulation of oxidative stress may influence the pathological transition from steatosis to steatohepatitis.

Inflammatory responses also play a critical role in the progression of steatohepatitis. Ly6C-positive monocytes are known to migrate to injured liver tissue and differentiate into inflammatory macrophages, thereby contributing to disease progression [32]. Activation of hepatic macrophages, including Kupffer cells, has been reported to promote sustained inflammatory responses and tissue damage [33]. In the present study, naringin treatment reduced inflammatory cell infiltration and decreased the expression of immune-related markers. In vivo experiments further demonstrated altered expression of inflammation- and macrophage-related genes, including *Adgre1*, *Ccl2*, and *Tnf*, suggesting that naringin may regulate hepatic inflammatory responses and monocyte recruitment. Network pharmacology analysis also identified inflammatory signaling–related targets such as PTGS2, which is consistent with the immune cell activation changes observed in this study. The simultaneous reduction in Ly6C-positive cell accumulation and Kupffer cell activation suggests that naringin may suppress not only the recruitment of inflammatory monocyte-derived macrophages but also the activation of resident hepatic immune cells.

Persistent inflammatory activation is closely associated with hepatic stellate cell activation, which represents a key mechanism driving fibrotic remodeling. Activation of hepatic stellate cells leads to excessive extracellular matrix accumulation and progressive fibrosis [34,35]. In the present study, naringin treatment attenuated fibrotic changes, suggesting that regulation of upstream processes such as lipid metabolism, oxidative stress, and inflammation may contribute to the suppression of fibrosis progression. In vivo experiments showed altered expression of fibrosis-related genes including *Timp1*, *Acta2*, and *Col3a1*, supporting the possibility that naringin affects hepatic stellate cell activation and extracellular matrix deposition. These findings are consistent with network pharmacology results identifying fibrosis-related targets such as SERPINE1, MMP2, and ACE, which are known to play important roles in extracellular matrix remodeling and fibrogenesis [36,37].

In addition to hepatic changes, alterations in hypothalamic microglial activity were observed, suggesting that steatohepatitis may involve regulatory mechanisms beyond the liver. Although the present results do not establish a direct causal relationship between hypothalamic changes and hepatic pathology, they support the possibility that metabolic liver disease involves bidirectional communication among peripheral metabolic organs, the immune system, and central regulatory pathways. Naringin is known to be metabolized by intestinal microbiota into its aglycone form, naringenin, which has higher bioavailability and biological activity. This metabolic conversion may allow the compound to reach systemic circulation more efficiently and influence not only hepatic processes but also peripheral immune responses and central regulatory mechanisms. Collectively, these findings suggest that naringin, via its metabolite naringenin, may influence both peripheral metabolic and inflammatory processes and central neuroinflammatory responses through a liver–brain axis, potentially contributing to the observed reduction in hypothalamic microglial activation [15].

Several limitations should be considered. First, the genes analyzed in the in vivo experiments were not limited to the targets predicted by network pharmacology but also included additional markers selected to reflect the pathophysiology of the MCD model. Therefore, the causal relationship between predicted targets and experimental findings requires further validation, particularly for the key hub targets identified through network pharmacology and molecular docking (e.g., PPARG, PTGS2, SERPINE1, and CASP3). Second, the present study primarily evaluated transcriptional and histological changes, and protein-level alterations or direct activation of signaling pathways were not assessed. Furthermore, future studies incorporating protein expression analyses (e.g., Western blot or ELISA) and pathway-specific functional experiments will be necessary to confirm the direct involvement of these targets. Third, the MCD diet model reproduces key features of steatohepatitis, including oxidative stress, inflammation, and fibrosis, but does not fully reflect obesity and insulin resistance commonly observed in human MASLD. Accordingly, additional studies using alternative metabolic models (e.g., high-fat or high-fructose diet-induced models) are warranted to further validate the metabolic effects of naringin. Fourth, the sample size in this study was limited, and some analyses may have been underpowered.

## 5. Conclusions

This study demonstrated that naringin exerts protective effects on multiple pathological processes in an MCD diet-induced model of metabolic dysfunction-associated steatohepatitis. Network pharmacology analysis predicted that naringin may interact with multiple targets related to lipid metabolism, oxidative stress, inflammatory responses, and fibrosis. In vivo experiments showed that naringin treatment improved serum liver enzyme levels, showed limited effects on lipid parameters, reduced hepatic lipid accumulation and oxidative stress, suppressed inflammatory cell infiltration, and attenuated fibrotic changes. In addition, changes in hypothalamic microglial activity were also observed.

These findings suggest that the action of naringin is not limited to a single pathway but may involve coordinated regulation of multiple pathological mechanisms involved in the progression of steatohepatitis.

Overall, naringin may represent a multitarget plant-derived candidate with therapeutic potential for complex immunometabolic diseases such as MASH. Further studies using additional metabolic models and protein-level analyses are required to clarify the precise molecular mechanisms and to evaluate its potential for clinical application.

## Figures and Tables

**Table 1:** Molecular docking results of naringin with representative MASH-related target proteins.

Gene	PDB ID	Binding Affinity (kcal/mol)
*PPARG*	5U46	−9.1
*ESR1*	5KCT	−7.9
*ALB*	4QN5	−9.4
*SREBF1*	1US0	−9.1
*SREBF1*	4KAK	−7.8
*FGF21*	5VAQ	−8.1
*PNPLA3*	3U8D	−9.3
*PNPLA3*	1R6J	−6.8
*CD36*	5LGD	−8.8
*CASP3*	6X8I	−9.4
*CASP6*	2H5I	−7.6
*CASP7*	4ZVQ	−8.7
*CASP8*	2VWR	−7.6
*PTGS2*	4PH9	−9.6
*CCL2*	3BKB	−8.3
*TNF*	3KQ0	−8.6
*TNF*	7OUB	−10.4
*IL6*	2OOQ	−8.3
*IL6*	3BCJ	−9
*IL6*	5MJ3	−8.9
*SERPINE1*	7AQF	−10.3
*MMP1*	3SHI	−8.7
*MMP2*	3AYU	−7.5
*ACE*	9J1E	−8.1
*TGFB1*	4X2G	−9.3

For certain targets, multiple PDB structures were used to account for structural variability.

## Data Availability

The original contributions presented in this study are included in the article. Further inquiries can be directed to the corresponding authors.

## Abbreviations

The following abbreviations are used in this manuscript:

ACTA2	Alpha-smooth muscle actin
ALT	Alanine aminotransferase
AST	Aspartate aminotransferase
CAT	Catalase
CCL2	C-C motif chemokine ligand 2
COL3A1	Collagen type III alpha 1 chain
CPT1A	Carnitine palmitoyltransferase 1A
CYP2E1	Cytochrome P450 2E1
FASN	Fatty acid synthase
GFAP	Glial fibrillary acidic protein
GO	Gene Ontology
Iba-1	Ionized calcium-binding adapter molecule 1
KEGG	Kyoto Encyclopedia of Genes and Genomes
Ly6C	Lymphocyte antigen 6 complex, locus C
MASLD	Metabolic dysfunction-associated steatotic liver disease
MASH	Metabolic dysfunction-associated steatohepatitis
MCD	Methionine- and choline-deficient diet
NAR	Naringin
PPARA	Peroxisome proliferator-activated receptor alpha
PPARG	Peroxisome proliferator-activated receptor gamma
SOD2	Superoxide dismutase 2
SREBF1	Sterol regulatory element-binding transcription factor 1
TGFB1	Transforming growth factor beta 1
TIMP1	Tissue inhibitor of metalloproteinase 1
TNF	Tumor necrosis factor
